# In Silico Trials of Prosthetic Valves Replicate Methodologies for Evaluating the Fatigue Life of Artificial Leaflets to Expand Beyond In Vitro Tests and Conventional Clinical Trials

**DOI:** 10.3390/biomedicines13051135

**Published:** 2025-05-07

**Authors:** Pengzhi Mao, Min Jin, Wei Li, Haitao Zhang, Haozheng Li, Shilong Li, Yuting Yang, Minjia Zhu, Yue Shi, Xuehuan Zhang, Duanduan Chen

**Affiliations:** 1 School of Medical Technology, Beijing Institute of Technology, Beijing 100081, China; pengzhi.mao@foxmail.com (P.M.); 3120226000@bit.edu.cn (H.L.); shilong_li@foxmail.com (S.L.); yuting_yyt@163.com (Y.Y.); 2 Department of Cardiac Surgery, Nanjing Drum Tower Hospital, Nanjing 210008, China; drjinmin@126.com (M.J.); haitao_zhang_pumc@163.com (H.Z.); 3 School of Mechanical Engineering, Beijing Institute of Technology, Beijing 100081, China; lliw@bit.edu.cn; 4 Graduate School, Peking Union Medical College & Chinese Academy of Medical Sciences, Beijing 100730, China; 5 School of Disaster and Emergency Medicine, Tianjin University, Tianjin 300072, China; minjia_16@tju.edu.cn; 6 Enlight Medical Technologies (Shanghai) Co., Ltd., Shanghai 201318, China; yue_shi@enlight-medical.com

**Keywords:** in silico trials, artificial leaflets, fatigue life, crack propagation

## Abstract

**Background**: Fatigue failure of artificial leaflets significantly limits the durability of prosthetic valves. However, the costs and complexities associated with in vitro testing and conventional clinical trials to investigate the fatigue life of leaflets are progressively escalating. In silico trials offer an alternative solution and validation pathway. This study presents in silico trials of prosthetic valves, along with methodologies incorporating nonlinear behaviors to evaluate the fatigue life of artificial leaflets. **Methods**: Three virtual patient models were established based on in vitro test and clinical trial data, and virtual surgeries and physiological homeostasis maintenance simulations were performed. These simulations modeled the hemodynamics of three virtual patients following transcatheter valve therapy to predict the service life and crack propagation of leaflets based on the fatigue damage assessment. **Results and Conclusions**: Compared to traditional trials, in silico trials enable a broader and more rapid investigation into factors related to leaflet damage. The fatigue life of the leaflets in two virtual patients with good implantation morphology exceeded 400 million cycles, meeting the requirements, while the fatigue life of a virtual patient with a shape fold in the leaflet was only 440,000 cycles. The fatigue life of the leaflets varied considerably with different implant morphologies. Postoperative balloon dilation positively enhanced fatigue life. Importantly, in silico trials yielded insights that are difficult or impossible to uncover through conventional experiments, such as the increased susceptibility of leaflets to fatigue damage under compressive loading.

## 1. Introduction

Heart valves are connective tissue that restrict blood backflow within the heart by opening and closing. The atrioventricular valves include the mitral and tricuspid valves, which are located between the atria and ventricles of the vertebrate heart, while the semilunar aortic and pulmonary valves are located at the roots of the aorta and pulmonary artery, respectively [1]. Valve stenosis and regurgitation occur when valve leaflets are unable to open and close normally due to rheumatism, calcification, inflammation, and other causes, producing abnormal hemodynamics within the heart. Annually, approximately 306,000 individuals worldwide die from rheumatic valve disease, with a median age of death among patients in low-income countries of 28.7 years [2]. Aortic valve disease accounts for 61% of all deaths from heart valve diseases [3].

Transcatheter valve therapies that use catheter-based techniques to repair valves or implant replacement prosthetic valves are minimally invasive, allow a rapid recovery, and are generally low risk [4]. Biologically derived prosthetic valves exhibit good biocompatibility, high similarity to native heart valves, and a low risk of thrombosis [5]. Compared to valve repair surgical procedures, transcatheter aortic valve implantation (TAVI) has reduced all-cause mortality by 43.7%, and transcatheter mitral valve repair (TMVR) has decreased the postoperative heart failure rate by 47.3% [6]. Despite the positive impact of transcatheter valve therapies on valve disease treatment, the limited durability of biologically derived prosthetic valves is estimated to be only 10–20 years [7,8]. Clinical retrospective reports have indicated that artificial leaflets may tear within 2 years of transcatheter valve therapies [9,10]. Premature failure of artificial leaflets can lead to functional degradation, calcification, and thrombosis of prosthetic valves, resulting in vascular obstruction, regurgitation, and even death [11]. Artificial leaflet tearing has been attributed to fatigue wear under long-term work cycles [12]. Ternacle [13] posited that the repetitive mechanical stress experienced by artificial leaflets during heart contraction and relaxation is the primary cause of fatigue wear and functional degradation. Abnormal hemodynamics caused by prosthetic valve dysfunction further leads to an uneven distribution of mechanical stress on the surface of artificial leaflets, accelerating fatigue wear. Noble [14] first identified fatigue damage areas in two types of biologically derived artificial leaflets after accelerated fatigue wear testing using digital image correlation technology, then conducted biaxial tensile tests on tissue samples from the areas of wear to assess changes in their mechanical properties, and finally observed the microstructural changes in the artificial leaflets using confocal microscopy and electron microscopy. The results indicated a positive correlation between the degree of fatigue microstructural damage and the decline in mechanical properties for both types of leaflets. To explore the relationship between fatigue wear and the fatigue life of artificial leaflets, Zhou [12], Zhang [15], and Martin [8,16] have all proposed theoretical frameworks for fatigue damage in idealized artificial leaflet models using finite element simulation of fatigue. The notable success of Self-Supervised Learning (SSL) in medical applications, demonstrating exceptional generalization capabilities and remarkable computational efficiency through autonomous extraction and prediction of fatigue-associated biomarkers, warrants particular attention. Nevertheless, critical limitations persist regarding its substantial dependence on large-scale unlabeled datasets for pre-training optimization and the inherent interpretability challenges in elucidating the physiological mechanisms underlying its predictive outcomes [17]. In silico trials have emerged as a novel validation pathway for the safety and efficacy of medical devices and disease diagnosis and treatment methods. Compared to conventional clinical trials, the advantages of in silico trials include the following [18]: (1) More validation results are obtained before initiating animal or clinical studies. (2) Trial cohorts can be expanded to include rare, extreme, or difficult-to-recruit patient phenotypes. (3) Two alternative treatment methods can be compared in the same virtual patient. (4) Device performance is evaluated under challenging physiological conditions. (5) The number of animals or clinical patients required for the research is reduced, thus embodying the 3R principles (replacement, reduction, refinement) to protect the rights of research subjects.

As evidenced by previous cases, although standardized in vitro testing can validate the baseline fatigue durability of artificial valve leaflets, it fails to account for fatigue life heterogeneity caused by anatomical and nonlinear hemodynamic variations across patients under real physiological conditions. Furthermore, due to the bulky nature and intermittent operation of clinical instruments following prolonged fatigue testing, there remains a critical deficiency in effective strategies for real-time monitoring of functional performance in artificial heart valves without spatiotemporal constraints [19]. This necessitates the development of patient-specific fatigue life prediction models as a critical advancement in optimizing prosthetic valve durability assessment. In this study, we investigated valvular diseases and the fatigue life of artificial leaflets by using in silico trials. Our proposed method explicitly accounts for both material nonlinearity and motion-induced nonlinear behavior in artificial leaflets, allowing us to broaden the scope of fatigue life predictions and conduct exploratory analyses of crack propagation beyond what is possible in clinical trials. Our proposed innovative fatigue life evaluation method overcomes the limitations of in vitro tests, traditional finite element analysis (FEA), and conventional clinical trials. We aimed to gain a more comprehensive and quick understanding of the fatigue mechanisms of artificial leaflets to enhance the overall lifespan of these devices and reduce the patient risk. This provides a more accurate and comprehensive reference for designing and selecting prosthetic valves. Furthermore, we investigated whether in silico trials could replicate the outcome of conventional clinical trials using independent virtual patients. We demonstrated the potential for in silico trials to facilitate exploratory experiments that are challenging to conduct within conventional clinical trials, thereby offering insights that were not previously possible to obtain.

## 2. Materials and Methods

### 2.1. Clinical Patient and Image Acquisition

This study was approved by the Medical Ethics Committee of Affiliated Nanjing Drum Tower Hospital (2024-390-02). Formal consents from the examined patients were obtained prior to the study. The investigation conformed to the principles outlined in the Declaration of Helsinki.

We used pre- and postoperative dynamic computed tomography angiography (CTA) data of a clinical patient who underwent a valve-in-valve (ViV) transcatheter aortic valve implantation (TAVI) procedure at the Drum Tower Hospital affiliated with Nanjing University (Nanjing, China). Intraoperative digital subtraction angiography data, postoperative echocardiographic imaging data, and aortic valve pressure gradient data were also used. The prosthetic valve implanted during surgery was a TaurusOne (PEIJIA Medical Ltd., Suzhou, China). The CTA datasets were acquired using a dual-source CT scanner (uCT 960+, United Imaging, Shanghai, China).

### 2.2. In Vitro Pulsatile Flow Tests

**Figure 1:**
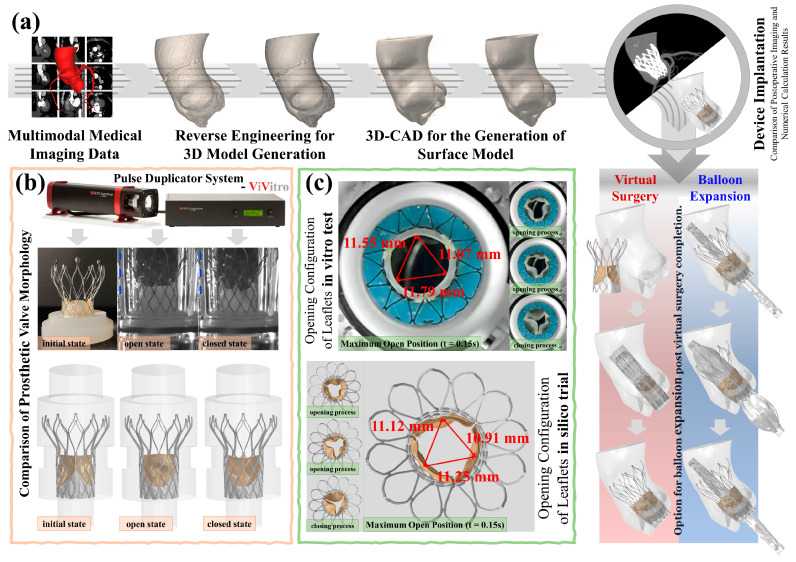
Virtual surgeries and in vitro pulsatile flow tests: (**a**) workflow of the prosthetic valve in silico trial; (**b**) a comparison of the prosthetic valve morphologies between the in silico trial and in vitro tests; (**c**) a comparison of the artificial leaflet opening configuration between the in silico trial and in vitro tests.

The TaurusOne was tested in vitro in a pulse duplicator system (ViVitro Labs Inc., Victoria, BC, Canada) (Figure 1b), operating at a rate of 70 beats per minute (bpm). The test chamber was filled with 37 °C distilled water to simulate the testing environment recommended by ISO 5840-3 [20]. A linear motor-driven plunger pump was used to precisely control the pressure values on both sides of the fluid according to the aortic valve pressure gradient curve of the clinical patient. A high-speed camera was used to capture slow-motion videos through a transparent chamber to observe the morphological movements of the artificial leaflets. The morphological parameters of the prosthetic valve were evaluated, including the maximum opening size of the leaflets, opening pattern, closing pattern, and the compressed configuration of the metal stent.

### 2.3. In Silico Trials of Prosthetic Valves

The overall in silico workflow comprised four main tasks: (1) processing images, (2) establishing virtual patient models and creating numerical models, (3) defining material properties and boundary conditions, and (4) performing numerical simulation analysis.

#### 2.3.1. Establishing Virtual Patient Models

We developed three virtual patient models, all of which shared identical model parameters and boundary conditions. The models differed in their data sources: virtual patient A was modeled based on data from in vitro pulsatile flow tests, while virtual patients B and C were modeled using data from the clinical patient. Additionally, virtual patient B underwent virtual balloon expansion, while virtual patient C did not.

For virtual patient A, we created a 1:1 scale fluid domain model of the pulse duplicator system test chamber and the TaurusOne valve. The TaurusOne model comprised a metal stent, three artificial leaflets, and the skirts of the prosthetic valve. For virtual patients B and C, the preoperative CTA data of the clinical patient underwent threshold segmentation to extract the region from the left ventricular outflow tract to the ascending aorta. Then, surface models of the aorta and the surgical frame were generated by reverse reconstruction. Subsequently, the aortic wall and surgical frame surfaces were smoothed and trimmed. An immersed fluid domain model and a three-dimensional balloon model were established using the three-dimensional surface data of the clinical patient’s aorta. The process for generating the virtual patient models is depicted in Figure 1a. The aortic wall, surgical frame, artificial leaflets, prosthetic valve skirt, and balloon were meshed using a mix of quadrilateral and triangular shell elements, modeled as S4R and S3R elements, respectively. The metallic stent was meshed with six-node triangular prism elements, modeled as C3D6 elements. The two immersed fluid domains were meshed with hexahedral elements and modeled as C3D8 elements.

#### 2.3.2. Material Properties

The metallic stent of the TaurusOne is fabricated from nitinol, and its superelastic and isotropic behaviors were simulated using the theoretical model proposed by Auricchio [21]. The specific material properties are detailed in Table 1. The artificial leaflets are fabricated from a hyperelastic material with nonlinear characteristics, and the material attributes were ascertained utilizing tensile testing methodologies [22] in our laboratory. Previous research demonstrated that the Mooney–Rivlin constitutive model [23], based on the strain energy density, can effectively describe this type of hyperelastic material. In this study, a special case of the Mooney–Rivlin constitutive equation with only one term of the strain energy density function, known as the neo-Hookean constitutive model, was used to describe the leaflet material [24]. The strain energy equation is given by

(1) $$U=C_{10}\left(I_{1}-3\right)+\frac{1}{D_{1}}{\left(J^{el}-1\right)}^{2}$$

where *U* represents the strain energy per unit reference volume, $I_{1}$ is the first strain invariant, $J^{el}$ is the total volume ratio, and $C_{10}$ and $D_{1}$ are parameters related to the initial shear modulus $\mu_{0}$ and Poisson’s ratio $\nu_{B}$ according to the following relationships

(2) $$C_{10}=\mu_{0}/2$$

(3) $$D_{1}=3(1-2\nu_{B})/\mu_{0}(\nu_{B}+1)$$

The material parameters for the leaflets were previously obtained from the mechanical testing of glutaraldehyde-treated bovine pericardium samples by Dong [25]. We used the Mie–Grüneisen equation of state to describe the dynamic response behavior of blood flow as

(4) $$P=C_{0}^{2}{\left(\frac{\rho }{\rho_{0}}\right)}^{s}+\left(s-1\right)u$$

where *P* denotes pressure, ρ is the compressed density, $\rho_{0}$ is the initial density, s is the Grüneisen parameter, u is the internal energy, and $C_{0}$ represents the speed of sound in the material under unstrained conditions. The parameters $C_{0}$ and s are critical as they together determine the behavior of the material’s equation of state.

#### 2.3.3. Virtual Surgeries

The aortic wall thickness could not be constructed directly from the CTA data, and therefore, a uniform thickness of 2 mm was assigned to the aortic walls by specifying the shell element thickness [26]. Using the TaurusOne product, the artificial leaflets were set to a uniform thickness of 0.3 mm. Full consideration was given to the interactions and self-contacts within each model by using a penalty contact algorithm, with a normal behavior set as “hard contact”. The specific virtual surgery process was modeled as follows (Figure 1a): (1) A cylindrical thin-walled shell was used to simulate the catheter sheath, with the thin-walled shell contracting inward to mimic the compression and loading process of the prosthetic valve. (2) The position and angle of the catheter sheath were adjusted to align its axis perpendicular to the aortic root plane, after which the constraint of the sheath on the prosthetic valve was removed. Because of the hyperelasticity and shape memory properties of the nitinol alloy, the self-expandable metal stent automatically deployed and anchored within the aortic wall and the surgical frame. (3) The balloon was compressed and inserted into the prosthetic valve, and then, a 1-MPa pressure load was applied within the balloon. The pressure was then released and the balloon was removed. The expansion of the balloon smoothed the artificial leaflets. During expansion, both ends of the balloon were maintained in a fixed position.

#### 2.3.4. Virtual Postoperative Physiological Maintenance Simulation

We simulated the postoperative motion of the artificial leaflets under various physiological conditions representing virtual patients using a two-way fluid–structure interaction (FSI) algorithm based on the coupled Euler–Lagrange (CEL) method. The CEL method has been demonstrated to effectively handle issues with large deformations in numerical simulations [27,28] and has been previously applied to the coupled calculations of heart valve kinematics and hemodynamics [29,30,31]. In the CEL approach, the entire computational domain is treated as a fluid domain, within which an immersed boundary is modeled [32,33]. The solid boundary is represented by a set of forces distributed over the fluid grid, and these forces are applied to the fluid grid through interpolation, thus influencing the fluid motion [34,35]. Ultimately, the solid displacement and velocity are updated based on the fluid flow results, and new solid boundary forces are calculated.

**Figure 2:**
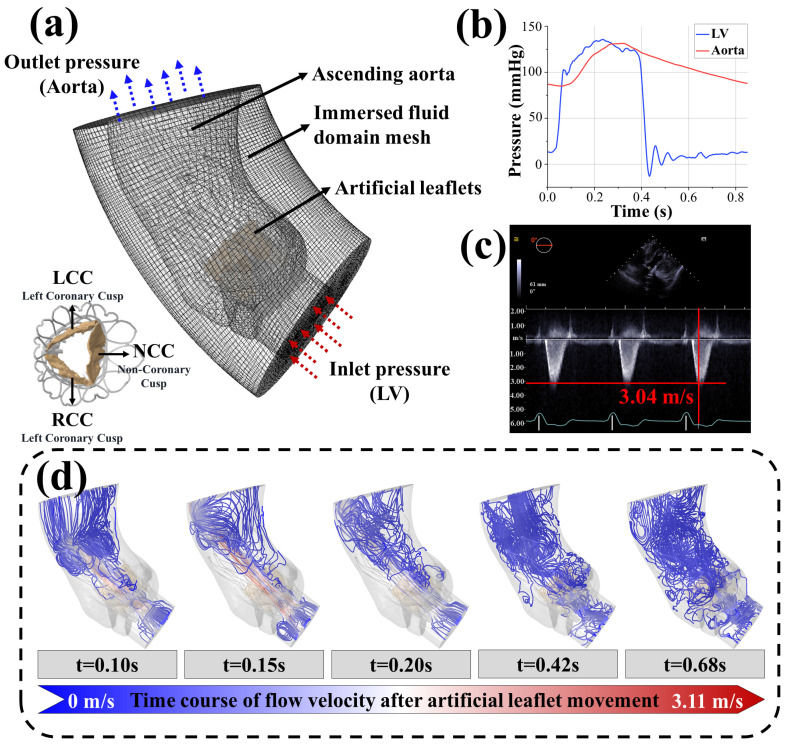
Virtual postoperative physiological maintenance simulation: (**a**) in silico trial models and boundary conditions, red arrows indicate the inflow end, and blue arrows indicate the outflow end.; (**b**) the aortic valve pressure gradient curve of the clinical patient; (**c**) postoperative echocardiography of the clinical patient; (**d**) typical flow velocity and flow field results of the in silico trial of virtual patient B at five time points during the cardiac cycle.

In the coupled computational model, all solid regions were fully immersed within the fluid domain and treated as static Eulerian grids (Figure 2a). Compared to the large deformations of the leaflets, the deformations of the aortic wall, surgical frame, metal stent, and skirts were negligible and, thus, were modeled as rigid bodies with all degrees of freedom fixed. Pressure loads equivalent to those obtained in the pulsatile flow experiments described in Section 2.2 were applied at the inlet and outlet of the aorta. Using pulsatile pressure helps to better simulate the behavior of valves under nonlinear dynamics [36]. The numerical model treated blood as an incompressible Newtonian fluid with an initial density equal to the compressed density. The blood density was 1.06 g/cm^3^ and the viscosity was 0.00365 Pa·s. The stress distribution results of the artificial leaflets obtained from the virtual surgery were used as the initial stress values for the coupled computational model, and the remaining boundary conditions were consistent with those of the virtual surgery. The analysis encompassed five cardiac cycles to eliminate errors caused by program initialization. Data from the initial cardiac cycles were discarded, and data analysis was performed on data collected during subsequent cardiac cycles. The output parameters included blood flow trajectories, flow velocities, valve leaflet morphology, and leaflet surface stresses.

### 2.4. Fatigue Life Prediction Model of the Artificial Leaflets

In the laboratory, Dong [25] conducted ultra-high-cyclic pulsatile loading stress testing on glutaraldehyde-treated bovine pericardium specimens, incorporating considerations of fatigue damage accumulation and permanent deformation during cyclic tensile loading to derive the material’s nonlinear fatigue S-N curve. This nonlinear S-N curve describes the number of pulsatile cycles that cause fatigue failure at a given stress level. Combining the nonlinear S-N curve with theories of fatigue damage accumulation [37], we proposed a method for calculating the fatigue life of leaflets. The formula for calculating the fatigue damage, Da, is given by

(5) $$Da=\frac{N}{N_{f}}$$

where *N* is the actual number of pulsatile cycles experienced, and $N_{f}$ is the number of pulsatile cycles required for fatigue failure at a specific stress level. The total cumulative damage is obtained by summing the damage from all cycles

(6) $${Da}_{total}=\sum {Da}_{i}=\sum \frac{N_{i}}{N_{fi}}$$

where *i* denotes the $i_{th}$ cycle. If the total cumulative damage ${Da}_{total}$ equals 1, the material is considered to have reached its fatigue life. Therefore, the total expected life *L* (unitless) can be inferred from the cumulative damage

(7) $$L=\frac{1}{{Da}_{total}}$$

Load cycles in the load spectrum are often non-pulsatile. Here, we unified the load form by applying the Gerber equation to convert all cycles to pulsatile cycles. By modifying the stress amplitude using the parabolic Gerber equation, the mean stress effect on the high-cycle fatigue strength of nonlinear materials can be described [38]. The correction equation is given as

(8) $$\frac{S_{a}}{S_{Nf}}+{\left(\frac{S_{m}}{S_{u}}\right)}^{2}=1$$

where $S_{u}$ is the nominal ultimate tensile strength of the material, $S_{a}$ is the alternating stress of each cycle segment, $S_{m}$ is the mean stress of each cycle segment, and $S_{Nf}$ is the fatigue strength of the material under fully pulsatile cycles.

The formula for calculating the alternating stress $S_{a}$ is

(9) $$S_{a}=\frac{S_{max}-S_{min}}{2}$$

And the formula for calculating the mean stress $S_{m}$ is

(10) $$S_{m}=\frac{S_{max}+S_{min}}{2}$$

where $S_{min}$ is the minimum stress and $S_{max}$ is the maximum stress for each cycle segment.

We consider stress concentration areas to be at a high risk for fatigue failure [8,12,15,16]. Here, the fatigue life at the maximum stress integration point was considered the total life of the artificial leaflets. Based on the above theory of fatigue life prediction, we first extracted the load spectrum of the integration point that corresponded to the maximum stress on the leaflet surface during a complete cardiac cycle. We then considered similar peaks in the load spectrum to be equal and averaged them, which simplified the load spectrum to reduce the computational effort. Using the rainflow counting method [39], we extracted all sub-cycles within the load spectrum and recorded the number of identical sub-cycles as n. During the actual calculation, we found that there were two types of cycles in the load spectrum: full cycles and half-cycles. A full cycle was defined as a complete cycle from the maximum stress to the minimum stress and then back to the maximum stress, while a half-cycle was a unidirectional stress change from a maximum to a minimum (or vice versa). Two half-cycles were considered equivalent to one full cycle. Considering the impact of half-cycles on fatigue damage, we counted all sub-cycles that appeared only once as 0.5.

According to the classical Wöhler theory and Basquin model theory [40,41,42], the relationship between $S_{Nf}$ and $N_{f}$ is

(11) $$S_{Nf}=\sigma_{f}{\left(2N_{f}\right)}^{\beta }$$

where $\sigma_{f}$ is the single-cycle fatigue strength (the maximum tensile true stress) limit of the artificial leaflet material, and β is the material fatigue life exponent, which describes the relationship between fatigue life and stress level and corresponds to the slope k of the high-cycle fatigue segment of Basquin-type S-N curves. The essence of the Basquin equation is an empirical formula, with parameters fitted based on Dong’s nonlinear fatigue experiments [25], and it does not rely on linear material assumptions.

By substituting Equation (7) into Equation (10), we calculated the number of pulsatile cycles $N_{fj}$ required to produce fatigue failure at the stress level of the $j_{th}$ sub-cycle. According to the cumulative damage theory, the cumulative damage at the point of the maximum stress over one load spectrum (one cardiac cycle) is described by

(12) $$Dai=\sum n_{ij}/N_{fj}$$

where $n_{ij}$ is the number of cycles of the $j_{th}$ sub-cycle in the $i_{th}$ pulsatile cycle.

In previous cardiac–aortic hemodynamic studies, we found that after excluding errors incurred during program initialization, the characterization of fluid dynamics tended to be consistent from the third cardiac cycle onward [43]. Therefore, we assumed that the load spectrum was the same in all subsequent cardiac cycles. By substituting Equation (12) into Equations (2) and (3), the total expected life *L* of the artificial leaflets is given by

(13) $$L=\frac{1}{\sum n_{j}/N_{fj}}$$

### 2.5. Crack Propagation Prediction Model of the Artificial Leaflets

We hypothesized that the region of the maximum stress concentration was the initial tear location and considered this area to be an elliptically shaped crack with a semi-major axis, *a* (mm), and a semi-minor axis, *b* (mm). The FSI calculations of the surface stress on the intact artificial leaflets were performed using the Glinka criterion [44], which is based on the strain energy density theory. Using this criterion, the maximum and minimum stress values around the crack were calculated. The Glinka criterion assumes that the strain energy density at the crack tip is nearly identical for both linear elastic and elastic-plastic notch behaviors when the plastic zone at the crack is surrounded by an elastic field. The stress is calculated at the crack according to the Glinka criterion

(14) $$\frac{\sigma^{2}}{E}+\frac{2\sigma }{e+1}{\left(\frac{\sigma }{K}\right)}^{\frac{1}{e}}=\frac{{\left(K_{t}S\right)}^{2}}{E}$$

where σ is the stress at the crack, *E* is the elastic modulus of the artificial leaflet material; *e* is the material’s work hardening exponent; *S* is the applied stress, which is the maximum stress value $S_{max}$ or minimum stress value $S_{min}$ from each full cycle in the load spectrum; and $K_{t}$ is the stress concentration factor, which is related only to the structure and shape of the crack, not to the material itself [45]. The formula for $K_{t}$ in the case of an elliptical crack is given by [46]

(15) $$K_{t}=1+2\frac{b}{a}$$

Using the classical Paris equation [47], we calculated the crack propagation length in each direction for each instance of the full-cycle loading using the following formula,

(16) $$\frac{d_{\alpha }}{d_{N}}=C{\left(∆K\right)}^{m}$$

where α is the crack propagation length, *C* is the crack growth rate of the artificial leaflet material, and *m* is the crack growth exponent of the material; both *C* and *m* are inherent material properties and can be determined by fitting cyclic tension mechanical tests of material samples. ∆*K* is the stress intensity factor range, calculated as

(17) $$∆K(\sigma max-\sigma min)\sqrt{\pi a}$$

The total crack propagation length under N full cycles of loading was determined by superposition [48], using the formula:

(18) $$\alpha_{N}=\alpha_{0}+\sum_{i=1}^{N}∆\alpha_{i}=\;\alpha_{0}+\sum_{i=1}^{N}f(∆K_{i})$$

where $\alpha_{0}$ is the initial crack length. When calculating the tear length along the major axis, the value of b is used; when calculating along the minor axis, the value of a is used.

## 3. Results

### 3.1. In Silico Process

We applied image processing, reverse engineering, virtual surgery techniques, and FSI algorithms to the TaurusOne project to construct virtual patient models for in silico trials, with potential use in in vitro testing and clinical trials. In silico testing closely represented real-world conditions regarding geometric complexity, material properties, boundary conditions, initial conditions, and input loads. To further investigate the impact of the optional surgical balloon dilatation procedure on the artificial leaflets, we modeled two types of virtual surgeries and calculated their corresponding virtual postoperative physiological homeostasis maintenance parameters. Balloon dilatation was performed only on virtual patient B. The results of the in vitro testing and virtual patient modeling are presented.

### 3.2. Accuracy Analysis of the In Silico Trials

The morphological analysis of prosthetic valves in virtual patients A, B, and C showed strong agreement between computational and experimental measurements (Figure 1b,c). For virtual patient A, the compressed stent configuration and leaflet kinematics (systolic opening/diastolic closing) matched clinical observations. Quantitative comparisons revealed:

i. Maximum leaflet displacement measurements during systole showed <1.5% variation between in vitro tests (11.07–11.79 mm) and computational results (10.91–11.25 mm, *P* > 0.05)
ii. Peak systolic flow velocity in virtual patient B (3.11 m/s) differed by <3% from postoperative echocardiography (3.04 m/s, Figure 2c,d)

These consistent morphological and hemodynamic outcomes support the validity of the computational framework’s parameter settings.

### 3.3. Variability in the Artificial Leaflet Fatigue Life

We conducted computational analyses of the motion of the artificial leaflets under three different conditions, corresponding to the three virtual patients. Fatigue life analysis was performed on the outer surface (bottom integration point) and inner surface (top integration point) at the locations of the maximum stress integration points. The calculated results are shown in Table 2.

**Figure 3:**
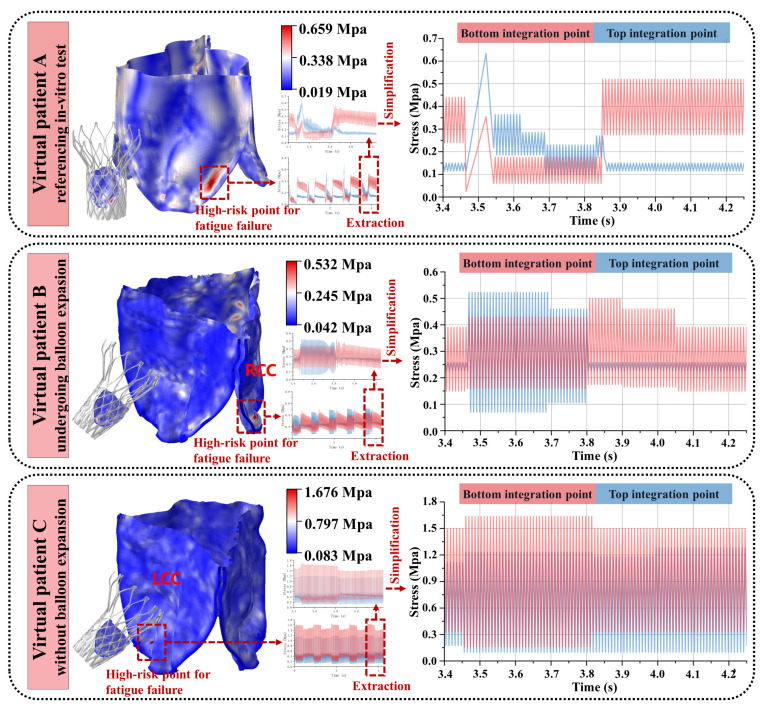
Load spectrums of the three virtual patients.

Computational analyses of artificial leaflet motion across three virtual patients revealed distinct fatigue patterns at maximum stress integration points. Post-initialization stabilization (cycles 3–5, Figure 3), consistent load patterns emerged with peak stresses occurring during systolic leaflet opening:

i. Patient A demonstrated 0.659 MPa maximum stress within an elongated concentration zone at the side fold of the leaflet base. Systolic blood flow induced characteristic root bending, generating outer surface compression (0.659 MPa), surpassing inner surface tension—a mechanical reversal observed during diastolic closure.
ii. Patient B exhibited 0.632 MPa circular stress localization at the right coronary cusp fold. Outer compressive stresses dominated systole (0.632 MPa vs. negligible inner tension < 0.05 MPa), with minimal diastolic variation (<5%) on the inner surface.
iii. Patient C developed 1.676 MPa acute stress focusing on the sharply folded left coronary cusp base. Persistent inner surface stresses prevailed throughout the cardiac cycle (peak: 1.676 MPa; mean/stress amplitude exceeding outer surface by 38–62%), maintaining dual-surface high-stress states.

Despite shared TaurusOne platform architecture (NMPA-approved 2021, validated against 4 × 10^8^ cycle requirement), fatigue life predictions diverged significantly. Clinical durability projections reflected implantation-specific biomechanics: Patient A’s configuration exceeded regulatory benchmarks with multi-decade longevity (>30 years at 70 bpm); Patient B maintained clinically acceptable performance (>12 years); Patient C’s pathological fold geometry precipitated catastrophic failure (<7 days)

This computational framework successfully captured critical failure mechanisms, including anatomical fold-dependent stress concentration morphologies, phase-specific stress reversal mechanisms (systolic compression—diastolic tension), and root bending kinematics influencing surface stress differentials. This variability originated from implantation-specific stress patterns and confirming model sensitivity to anatomical deployment conditions.

## 4. Discussion

To ensure that in silico trials can be reliably compared to conventional clinical trials, three principles must be adhered to [18,49]: (1) Each virtual patient must exhibit high fidelity relative to actual clinical trial patient data. (2) The virtual endpoints should reflect the true conditions of the corresponding endpoints within the clinical trial cohort. (3) The conclusions derived from the effects observed in both virtual and clinical patient samples, when the sample size is sufficiently large, must remain consistent. The reliability of computational models, such as those in virtual surgery and when using FSI-CEL and material property determination methods, has been adequately validated in previous research [8,12,15,16,29,30,31]. In this study, the virtual patient models were constructed using CTA imaging data of a clinical patient and data from a realistic and specific pulse duplicator device, and the computational in silico trial setup for left ventricular and aortic pressure curves was derived from actual intraoperative pressure measurement data. The morphological results of the artificial leaflets and the calculated blood flow velocity also exhibited high consistency with actual conditions. The accuracy of the proposed artificial valve leaflet fatigue life evaluation method and its results were fully confirmed and are presented in the Results Section. We demonstrated that similar quantitative conclusions regarding the fatigue life of artificial valve leaflets can be drawn from both in silico trials and clinical trials.

**Figure 4:**
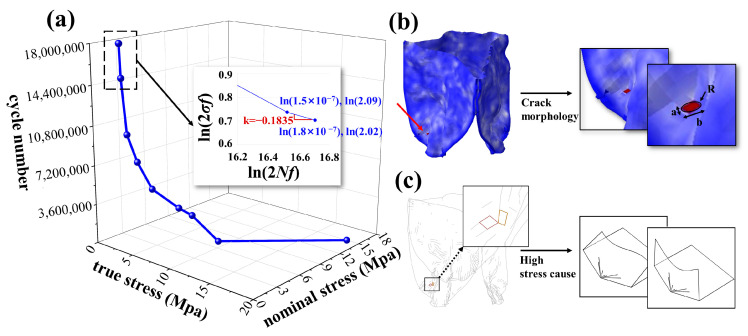
Analysis of fatigue life results: (**a**) The S-N curve of the artificial leaflet material, with the two horizontal axes representing true stress and nominal stress, respectively, and the vertical axis indicating the number of cycles. The Basquin curve plots the logarithms of 2$N_{f}$ and $\sigma_{f}$ on the horizontal and vertical axes, respectively. (**b**) It is hypothesized that the region of the maximum stress concentration serves as the initial site of tearing, and this area is modeled as an elliptical crack. (**c**) In the wireframe diagram of the artificial leaflet, it can be observed that the leaflet surface is compressed to form a sharp angle, akin to the sharp angle produced when paper is folded.

The early safety and efficacy of TaurusOne have been confirmed [50], but there is a lack of long-term follow-up data, particularly regarding the fatigue performance of the leaflets. Studies specifically investigating the degree of artificial leaflet damage during postoperative follow-up are particularly scarce. In our fatigue life prediction model for artificial leaflets, the fatigue lives of virtual patients A, B, and C were 4.58 billion, 458 million, and 440,000 cycles, respectively. Although the results for virtual patient A fulfilled the normal operational cycles for valve function within a typical human lifespan [1], the durability of the same prosthetic valve in virtual patients B and C varied significantly, which may be related to the distorted morphology of the artificial leaflets throughout their motion. Moreover, it is conceivable that different surgical maneuvers in clinical procedures may optimize or reduce the service life of the leaflets; for example, repeated repositioning and recapturing might cause microscopic leaflet damage, reducing fatigue life, and potentially create a pre-thrombogenic environment [51]. In this study, the only difference between virtual patients B and C was the use of balloon dilatation after the prosthetic valve implantation. The valves of both virtual patients exhibited similar and favorable postoperative hemodynamics, but during the implantation process of virtual patient C, the leaflet surface was compressed to form a sharp angle (Figure 4c), resulting in an abnormal and non-self-recoverable fold that generated extreme stress during leaflet cycling. In contrast, the sharp angle was smoothed in virtual patient B through balloon dilatation, significantly optimizing the leaflet’s service life. Therefore, we suggest that favorable hemodynamics are not significantly correlated with the failure probability of artificial leaflets [51], and that balloon dilatation has a positive effect on the fatigue life of self-expanding prosthetic valves. In a long-term follow-up study of the Boston LOTUS prosthetic valve [51], despite successful surgical outcomes, 51% of the devices that failed to function normally within 30 days were due to substandard valve performance. Thus, bridging this knowledge gap in preoperative planning and avoiding potential risks through virtual computation is crucial to ensuring favorable outcomes for TaurusOne recipients.

In the load spectrums of virtual patients A and B (Figure 3), compressive stresses consistently exceeded tensile stresses. A fatigue simulation study on prosthetic valve artificial leaflets [8] reported that leaflets are more susceptible to fatigue damage under compressive loading. A previous study indicated that glutaraldehyde-treated bovine pericardium is not entirely “immunologically inert”, and residual animal antigens may elicit a cellular immune response, leading to tissue degeneration [14]. Additionally, pressure promotes cellular fibrosis, resulting in leaflet contraction and calcification, which exacerbates cyclic fatigue damage [12,52]. In addition to tensile and bending stresses, shear stress is also a significant contributor to the mechanical fatigue and failure of leaflets [5,53]. Compared to the inner surface, the outer surface of the leaflets, which connects to the aortic wall to form sinusoidal structures, is more prone to both blood stasis and turbulent flow within the sinuses, thereby increasing shear stress on the outer surface [1]. Oscillating blood flow within the sinuses also increases the oscillatory shear stress on the outer surface [1]. The directional guidance of cellular migration by shear stress, as observed in vascularized microfluidic models, parallels its role in inducing fatigue crack initiation at high-stress regions of prosthetic leaflets, where cyclic mechanical loading accelerates material degradation, which, in turn, significantly reduces the fatigue life of the prosthetic leaflets [54]. Moreover, we observed that blood stasis in the sinuses was more likely to occur during diastole than during systole (Figure 2d), with a prolonged blood residence time in the sinuses potentially facilitating calcific material deposits on the outer surface [55], thereby reducing the fatigue life of the leaflets. Therefore, to increase the service life of current artificial leaflets, we suggest that the thickness of the material at the root where the leaflets connect to the metal stent should be increased in soft-material leaflets to enhance their bending stiffness [5]. The heterogeneous soft layer material exhibits a fracture behavior that is not sensitive to defect size [56], suggesting that the introduction of a layered structure in the design of artificial leaflets may alleviate local stress concentration and enhance fatigue life. Furthermore, in addition to using dialdehyde compounds for tissue crosslinking and sterilization, decellularization techniques should be used to remove cellular components from the tissue, thus preserving the extracellular matrix structure, further reducing immunogenicity, and mitigating issues with tissue fibrosis and calcification deposition [12]. The composite grafts incorporating acellular elastin demonstrated superior patency and mechanical strength in porcine models, indicating that elastin integration may further mitigate thrombogenesis on tissue-engineered artificial valve leaflet surfaces and enhance fatigue resistance [57].

A key advantage of in silico trials is the ease with which exploratory experiments can be undertaken as these are pivotal for generating novel insights and formulating new hypotheses. In in vitro crack propagation testing, the observed hysteresis and operational challenges can lead to significant errors in the measurement of crack dimensions. As a destructive testing method, clinical assessment is inherently limited in its capacity for observing and predicting crack propagation. In contrast to in vitro tests [12], the in silico trials in this study enabled predictions of crack propagation trends in artificial leaflets using our algorithm, with the propagation rate post-crack initiation measured with precision in millimeters and cardiac cycles. FEA has been used to simulate crack propagation in biological soft tissues [58,59,60,61]; however, due to the complex multi-physics environment at the aortic valve location and intricate nonlinear material fracture issues, the current FEA computational capabilities are insufficient. It is noteworthy that quantum computing holds the potential to accelerate microstructure simulations of materials in FEA or optimize damage parameters, yet this necessitates the development of quantum mechanical models specifically adapted for fatigue analysis. However, realizing this potential will require advancements in quantum hardware to achieve the high-precision qubits necessary for such complex multi-scale modeling, which may ultimately enable accelerated computational capabilities through this quantum-enhanced approach [62]. In studies on simpler biological soft tissues, such as cartilage [58] and dental tissues [59], the time required for FEA computations was no less than 72 h, whereas our crack propagation behavior prediction algorithm had a response time of less than 10 s.

## 5. Limitations

This study has several limitations. First, the small sample size of both virtual patient models and clinical cases, constrained by high computational costs and limited clinical data availability, may affect the generalizability of the findings. Although the computational results were validated against postoperative dynamic CTA and ultrasound imaging, the absence of multi-modal data (e.g., 4D-Flow and Cine imaging) and the short follow-up period of TaurusOne valve recipients preclude longitudinal comparisons of fatigue failure in real-world scenarios. Second, the assumption of blood as an incompressible Newtonian fluid, while commonly adopted in similar studies [29,30,31,59], might oversimplify hemodynamic effects and leaflet stress distribution by neglecting non-Newtonian behavior. Additionally, biologically coupled factors such as thrombosis and calcification, which could critically influence leaflet fatigue and crack propagation, were not incorporated into the analysis. Finally, the precision of crack propagation predictions remains constrained by incomplete model parameters, necessitating further refinement through multi-scale experimental data integration. Despite these limitations, this work establishes a conceptual foundation for mechanistic analyses of prosthetic valve fatigue.

## 6. Conclusions

In conclusion, this study demonstrated the application of advanced modeling and simulation techniques to analyze the kinematics of artificial leaflets under the nonlinear hemodynamic loading, introduced a novel prediction algorithm incorporating nonlinear material responses (via Gerber correction and Basquin parameters) for assessing the fatigue life of artificial leaflets under various conditions and configurations, and presented an efficient crack propagation prediction algorithm. The key findings are as follows: (1) In silico trials of prosthetic valves can provide additional insights into the fatigue failure of patients’ artificial leaflets, which are typically not obtainable from conventional clinical trials. (2) The fatigue life of artificial leaflets varied significantly under different implant configurations, and post-implantation balloon dilatation of prosthetic valves ameliorated adverse configurations of the artificial leaflets, thereby increasing the overall fatigue life. (3) During the opening and closing cycles, the roots of artificial leaflets experience high stresses and are prone to fatigue damage under compressive loads. Increasing the thickness of the roots of artificial leaflets during the manufacturing process can enhance their bending stiffness and increase their fatigue life. At the current stage of research, due to the inherent limitation of obtaining certain parameters, we have not yet been able to conduct precise numerical calculations of crack propagation in virtual patients. Despite these limitations, we maintain a strong confidence in the accuracy of our algorithms and their potential applicability. For future research, we plan to collect a broader range of data to further validate and optimize our algorithms.

## Figures and Tables

**Table 1:** Material properties.

Parameter	Metal Stent		Skirts	Artificial Leaflets	Balloon
Density, g/cm^3^	7.45		1.38	1.06	1.01
Poisson’s ratio	0.33		0.4	-	0.4
Young’s modulus, MPa	Austenite:	53,875	1000	2	1100
	Martensite:	29,600			
Transformation strain	0.055		-	-	-

**Table 2:** Predicted fatigue life of the artificial leaflets of three virtual patients.

Patient	Integration Point	Fatigue Life, Cycles
Virtual patient A	Top	5.65 × 10^9^
	Bottom	4.58 × 10^9^
Virtual patient B	Top	4.58 × 10^8^
	Bottom	2.02 × 10^9^
Virtual patient C	Top	9.30 × 10^5^
	Bottom	4.40 × 10^5^

## Data Availability

Data will be made available upon request.

## Abbreviations

The following abbreviations are used in this manuscript:

CEL	Coupled Euler-Lagrange
CTA	Computed tomography angiography
FEA	Finite element analysis
FSI	Fluid–structure interaction
ISO	International Organization for Standardization
LCC	Left coronary cusp
NCC	Non-coronary cusp
NMPA	National Medical Products Administration
NISQ	Noisy intermediate-scale quantum
RCC	Right coronary cusp
S-N curve	Stress–number curve
TAVI	Transcatheter aortic valve implantation
TMVR	Transcatheter mitral valve repair
